# Notalgia paresthetica: clinical features, radiological evaluation, and a novel therapeutic option

**DOI:** 10.1186/s12883-020-01773-6

**Published:** 2020-05-16

**Authors:** Cevriye Mülkoğlu, Barış Nacır

**Affiliations:** Department of Physical Medicine and Rehabilitation, Health Sciences University Ankara Training and Research Hospital, Ulucanlar street, Ankara, Turkey

**Keywords:** Notalgia paresthetica, Dorsalgia, Itching, Lidocaine, Spine

## Abstract

**Background/objective:**

Notalgia paresthetica (NP) is a sensory neuropathy characterized by localized pruritus and pain, presenting with or without a well-circumscribed hyperpigmented patch in the upper back. Abnormal sensations, such as burning, numbness, and paresthesia are often present in patients with NP. In this study, we clinically and radiologically analyzed patients with NP. The literature contains studies describing lidocaine treatments involving intravenous and topical applications for NP. We also investigated the effect of intradermal lidocaine injection on patients with NP.

**Methods:**

A total of 80 patients (45 patients with NP and 35 suffering from dorsalgia without NP) were included in the study. The age, gender and body mass index (BMI) of the patients, and the characteristics of their symptoms were recorded. The severity of pain and pruritus was assessed by the Visual Analog Scale (VAS). Radiography and magnetic resonance imaging of the spine were performed. In this study, we intradermally administered lidocaine diluted with saline into the upper back over three sessions. 1 cc 2% lidocaine was diluted with 5 cc 0.9% saline, and a total of 6 cc lidocaine mixture was obtained. The injection was performed locally at 1-cm intervals around the hyperpigmented patch and segmentally along the C2-T6 spinous processes. These patients were called for a follow-up at the second and fourth weeks and third month.

**Results:**

There was no statistically significant difference between the two groups in terms of age, BMI, VAS-pain score, and duration of symptoms (*p* > 0.05 for all). Forty-six cervical and/or thoracic degenerative changes or herniated nucleus pulposus (HNP) were detected in patients with NP. There was a significantly higher number of HNP at the C6–7 segment and cervical degenerative changes in the NP group (*p* < 0.05). The VAS-pain and VAS-pruritus scores were significantly decreased at all follow-up sessions, and improvement was sustained by lidocaine up to the third month.

**Conclusion:**

Cervical degenerative changes and HNP of the C6–7 segment seem to be contributing factors for NP. Local lidocaine can be effective for pain relief and pruritus in NP.

## Background

Notalgia paresthetica (NP) is characterized by localized chronic pruritus medial or inferior to the scapulae with or without an associated hyperpigmented macule. It is believed that NP is relatively common but perhaps underdiagnosed [1]. NP can exacerbate from time to time and lasts for months to years. It is widely accepted that NP is a sensory neuropathy which occurs as a result of the alteration of the cutaneous branches of the posterior rami, especially the upper branches of the T2-T6 spinal nerves [2, 3].

**Fig. 1:**
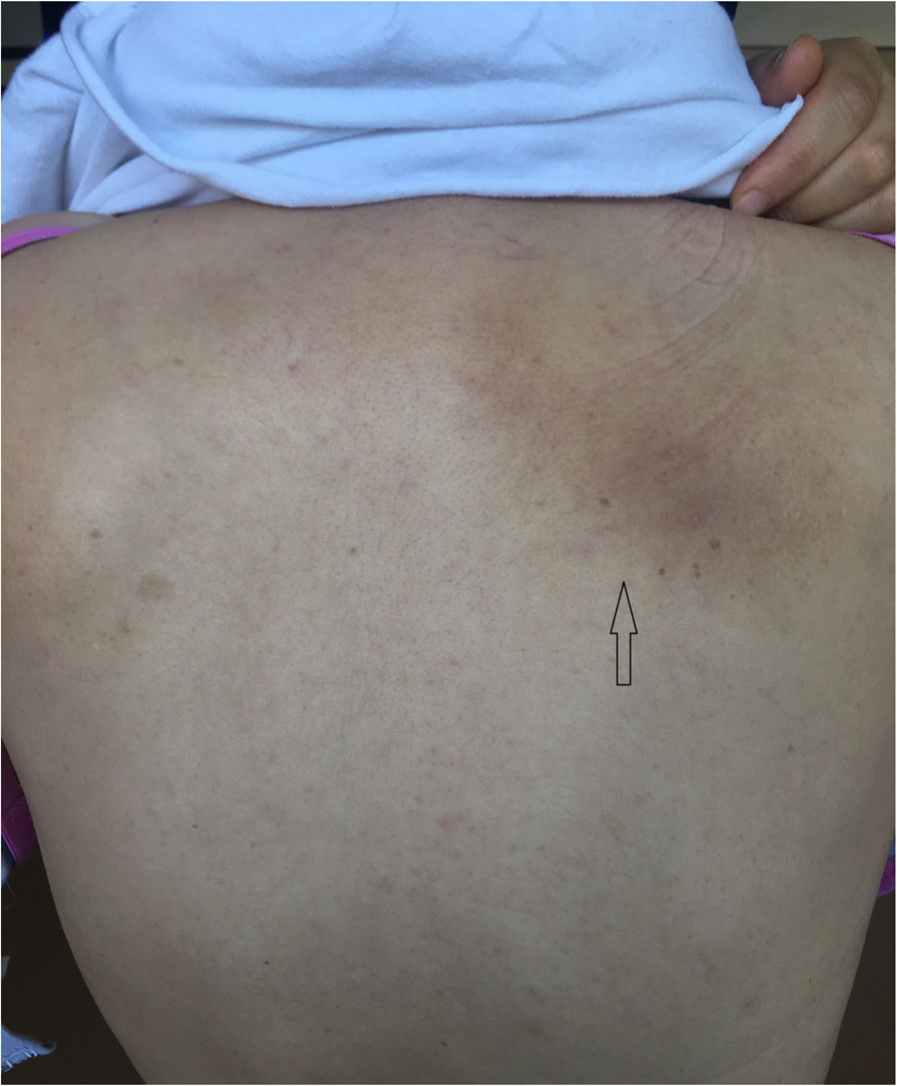
The arrow shows unilateral hyperpigmented macule in the medio-inferior scapulae

Although the etiology of NP has not yet been completely elucidated, degenerative changes of the cervical spine are considered to be associated with its pathogenesis [4, 5]. NP mostly occurs in women aged 54 to 62 years [6]. The condition is usually unilateral and rarely bilateral. Clinical symptoms in NP vary, including pain, burning, coldness, pruritus, numbness, tingling, paresthesia, allodynia, hyperalgesia, and hypoesthesia. Hypo/hyperpigmented well-circumscribed patches, macules, and hyperkeratosis may be observed secondary to scratching on the mid-upper portion of the back associated with the distribution of the T2-T6 dermatome [3, 7] (Fig. 1). Most studies suggested a thoracic polyradiculopathy due to spinal nerve entrapment is the primary etiology for pruritus. Another attributed factor is the anatomical right angle of sensory nerve fibers penetration through the multifidus muscle [3]. Muscle strain and spasm can cause these nerve fibers to impingement.

There are no primary cutaneous lesions in NP, these lesions are just secondary to chronic scratching and rubbing [8, 9]. Although the occurrence of NP is not rare, most cases are overlooked; therefore, it is often undiagnosed. The studies performed to date provide only limited data concerning the clinical and radiological findings of NP.

The treatment of NP is multidisciplinary, including topical agents (intralesional steroids, botulinum toxin A, capsaicin, and lidocaine), systemic drug treatments (gabapentin, oxcarbazepine, and amitriptyline) and physical therapy methods (TENS, cervical traction, exercise, and manipulation) [2, 3, 10]. In the literature, there are studies describing lidocaine treatments for NP. Cruz et al. reported a 39-years-old woman diagnosed with NP and treated by the daily application of topical lidocaine patches on the symptomatic region and exercises (postural corrective exercises, scapular muscle strengthening, and pectoral muscle stretching). The patient was mentioned that if she stopped using the lidocaine patches, the symptoms reappeared, at 2 weeks after the first examination. Three months later, she presented with complete symptom relief without the need for topical lidocaine treatment. The patient was symptom-free at the seventh-month follow-up [11]. Chtompel et al. presented a 50-year-old female with spinal cord injury. The patient was diagnosed with NP and treated with intravenous lidocaine for the management of NP. Three infusions of lidocaine at two-week intervals at a dose of 1 mg/kg bolus followed by 4 mg/kg infusion over 1 h were administered. Following the first two infusions, neuropathic pain was not relieved; however, significant relief of pruritus was observed [2]. We also previously reported a 73-year-old male case with NP treated with therapeutic lidocaine injections. In that case, we locally administered lidocaine into the upper back to relieve pain and obtained successful results related to neuropathic back pain. At the second-week follow-up, the VAS score of the patient was observed to have decreased from 7 to 1 [12].

The primary aim of this study was to investigate the clinical findings and localization of damage radiologically in patients with NP. Secondly, we aimed to evaluate the effect of therapeutic lidocaine injections on NP patients.

## Methods

In this cross-sectional study, a total of 80 patients who presented to our outpatient clinic between August 2018 and June 2019 were included in this study. Forty-five patients diagnosed with NP were recruited for the NP group and 35 who had dorsalgia without NP were included in the control group. Patients under 18 years of age, those with chronic inflammatory, infectious, neurological, psychiatric, rheumatologic and malignant diseases, local or diffuse other skin diseases, and those with history of trauma to the cervical and/or thoracic spine were excluded.

The study was approved by the local ethics committee and was conducted in accordance with the ethical standards specified in the 1964 Declaration of Helsinki and its later amendments. The patients were informed by the researchers about the aim of the study and the confidentiality of their personal information. The informed consent was obtained from all participants.

**Fig. 2:**
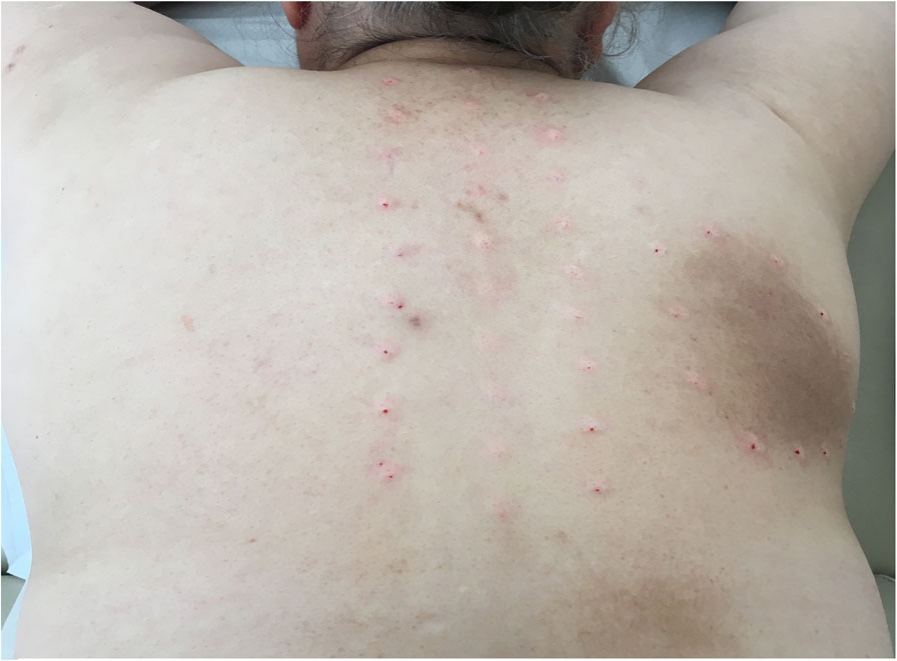
Lidocaine mixture diluted with saline was intradermally administered at 1-cm intervals around the hyperpigmented patch and along the C2-T6 spinous processes

The patients’ age, gender, weight and height were recorded. The body mass index (BMI) was calculated in kg/cm^2^. The characteristics and duration of the symptoms and dermatomal localization of the lesions were noted. The presence of spinal trauma history was questioned, and the patients with such history were not included in the study. The visual analogue scale (VAS) was used to assess the severity of current pain and pruritus based on a chart numbered from 0 (no symptoms) to 10 (maximum severity). All patients were queried for the localization of the complaints, including pruritus, pain, hyperpigmented skin lesions, and paresthesia. The diagnosis of NP was made by the same clinician based on the physical examination findings and medical history. The participants also underwent a neurologic examination of the spine and the extremities for motor and sensorial functions. The radiographic assessment with anteroposterior/lateral X-rays and magnetic resonance imaging (MRI) of the cervical and thoracic spine was completed for all participants, and the results were interpreted by another researcher blinded to the physical examination findings of the patients. The MRI findings of the cervical and thoracic spine, such as herniated nucleus pulposus (HNP), spinal stenosis, and degenerative changes were noted. After the diagnosis of NP and physical examination, therapeutic lidocaine injections were planned for the 22 patients in the NP group. Following the patients’ informed written consent, the clothes on the upper part of the body were removed. The patients were placed on the examination table in the prone position and asked to clench both hands and put their forehead on their hands and relax. The spinous processes between C2 and T6 and the right-left 2 cm lateral of these processes were marked with a pencil. Skin antisepsis was provided locally with chlorhexidine alcohol. Lidocaine solution diluted with saline was prepared. For this, 2% lidocaine ampoule and 0.9% saline were used. 1 cc lidocaine was diluted with 5 cc saline, and a total of 6 cc mixture was obtained. The diluted lidocaine mixture was intradermally administered at a dose of approximately 0.2–0.3 cc from each marked point. Small bumps appeared on the skin. The injection was performed locally at 1-cm intervals around the hyperpigmented patch and segmentally along the C2-T6 spinous processes (Fig. 2). After the first application, the injections were repeated at the second and fourth weeks, taking the total number of sessions to three. Topical capsaicin was prescribed to the remaining patients in the NP group. All of the patients were called for a follow-up at the second and fourth weeks and third month. The VAS-pain and VAS-pruritus scores were assessed at these visits.

### Statistical analysis

Statistical analyses were conducted using the Statistical Package for the Social Sciences (SSPS) software (SPPS Inc., Chicago, USA), version 21.0. The Shapiro-Wilk test was used to evaluate the normality of data distribution. The normally distributed data were presented as mean ± standard deviation (SD) and for the data without normal distribution, the median (minimum-maximum) and interquartile range (IQR) values were used. The categorical variables were given as numbers (n) and frequency rates (%). For the comparison of the paired groups, the independent samples t-test (normal distribution) and the Mann-Whitney U test (non-normal distribution) were used for the quantitative data. For non-normally distributed variables, the Friedman’s test was conducted. The relationship between the non-normally distributed variables was assessed with the Spearman correlation analysis. The Pearson chi-square test was used to test the differences in rates. *p* < 0.05 was considered to be statistically significant.

## Results

In this study, a total of 80 patients (45 with NP and 35 suffering from dorsalgia without NP) were assessed. The majority of the patients in the NP group were female (*n* = 39; 87%). In the control group, there were 21 (60%) females and 14 (40%) males. The number of the females in the NP group was significantly higher than those in the control group (*p* < 0.001). The mean age of the patients was 54.7 ± 12.2 (range 27–77) years for the NP group and 56.8 ± 10.4 years for the control group. The study groups were similar in terms of age, BMI, VAS-pain score, and duration of symptoms (*p* > 0.05 for all).

The characteristic of pain was burning in 22 patients (49%), pricking in 14 patients (31%), tingling in seven patients (16%), and coldness in two patients (4%) in the NP group. The complaints were localized around the right scapulae in 18 patients (40%), on the left side in 24 patients (53%) and bilateral in three patients (7%). Intense itching was present on the mid-upper back in all of the patients. Hypoesthesia was present in 18 patients (40%) and hyperesthesia in three (7%), while 20 (44%) patients described paresthesia in the affected area. A typical hyperpigmented unilateral patch around the medio-inferior scapulae was observed in 34 (76%) patients (Fig. 1). Only three patients had bilateral hyperchromic patches. Eight patients (18%) had no skin lesions on the back. The demographic and clinical characteristics of the participants are summarized in Table 1.

**Table 1:** Clinical and demographic characteristics of the patients with NP and in control group

	NP group (n:45)	Control group (n:35)	*P* value
Female (n)(%)	39 (87%)	21 (60%)	**< 0.001**
Male (n) (%)	6 (13%)	14 (40%)	
Age (years) (mean ± SD) (min-max)	54.7 ± 12.2 (27–77)	56.8 ± 10.4	0.64
BMI (kg/m^2^) (mean ± SD)	29.9 ± 1.8	27.8 ± 2.0	0.39
Duration of Symptoms (months)(Median) (min-max)(IQR)	12 (1–240) (33)	10 (2–190) (29)	0.28
VAS-pain (Median) (min-max)(IQR)	8 (5–10) (2)	7 (3–9)(2)	0.55
VAS-pruritus (Median) (min-max)(IQR)	6 (0–9) (7)	–	
Dermatomal Localization (n)(%)		–	
Right	18 (40%)		
Left	24 (53%)		
Bilateral	3 (7%)		
Clinical findings (n)(%)		–	
Pruritus	45 (100%)		
Burning	22 (49%)		
Paresthesia	20 (44%)		
Hypoesthesia	18 (40%)		
Prickling	14 (31%)		
Tingling	7 (16%)		
Hyperesthesia	3 (7%)		
Coldness	2 (4%)		

Bold *p* values show significance. Statistical significance was set at *p* < 0.05

*NP* Notalgia paresthetica, *n* Number, *BMI* Body mass index, *VAS* Visual analogue scale, *SD* Standard deviation, *IQR* Interquartile range

We did not find any statistically significant correlation between BMI and the duration of NP (*r* = 0.1, *p* = 0.38). There was also no statistically significant correlation between gender and VAS-pain scores (*r* = − 0.2, *p* = 0.12) in the NP group.

The radiological evaluation revealed 46 spinal pathologies, such as degenerative changes of the vertebrae, spinal stenosis, and HNP in 39 patients (87%) in the NP group. Ten of these patients had degenerative changes and spinal stenosis in the cervical spine and nine had degenerative changes or HNP in the thoracic segments. There were a total of 32 HNPs on the cervical and thoracic spine. Thirteen of 32 HNP were on the C6–7 segment (29%). There were nine HNP on the C5–6 segment (20%). The HNP of the thoracic segments were present in only five patients. In the control group, 21 patients had a total of 25 cervical and thoracic spinal pathologies. The number of HNP on the C6–7 segment was significantly higher in the NP group than in the control group. The remaining radiological findings of the control group were similar to the NP group (*p* > 0.05). The radiological findings of the participants are shown in Table 2.

**Table 2:** The cervical and thoracic radiological findings of the participants

Spinal pathology	NP group (n:39)	Control group (n:21)	*P* value
C3–4 HNP, C4–5 HNP (n)(%)	5 (11%)	3 (9%)	0.19
C5–6 HNP (n)(%)	9 (20%)	5 (14%)	0.06
C6–7 HNP (n)(%)	13 (29%)	6 (17%)	**0.02**
Cervical degenerative changes, spinal stenosis (n)(%)	10 (22%)	4 (11%)	**0.04**
Thoracic HNP	5 (11%)	4 (11%)	0.70
Thoracic degenerative changes (n)(%)	4 (9%)	3 (9%)	0.85

*NP* Notalgia paresthetica, *n* Number, *HNP* Herniated nucleus pulposus; Bold *p* values show significance. Statistical significance was set at *p* < 0.05

We planned local lidocaine injections for 22 patients. Since 2 patients did not come to the second injection, they were excluded from the study and the evaluation was made on 20 patients. After the third session of lidocaine injection, the VAS-pain and VAS-pruritus scores were significantly decreased at the second- and fourth-week follow-up. We did not observe any adverse effect due to lidocaine at the follow-up visits at the second and fourth weeks. The relief of pain and pruritus continued for up to 3 months. The results of lidocaine treatment are summarized in Table 3.

**Table 3:** The results of therapeutic lidocaine application on patients with notalgia paresthetica

	Patients (n:20)
The median VAS-pain score	
0.day	8 (5–9)
2nd week	4 (3–6)
4th week	2 (2–5)
	***p*** **< 0.001**
After 3 months	2 (1–4)
The median VAS-pruritus score	
0.day	7 (5–8)
2nd week	5 (3–6)
4th week	1 (0–4)
	***p*** **< 0.001**
After 3 months	1 (0–3)

*VAS* Visual analog scale, *n* Number; Bold *p* values show significance. Statistical significance was set at *p* < 0.05

## Discussion

NP was first described by a Russian neurologist Astwazaturow in 1934. The term notalgia is derived from notos (back) and algos (pain). NP is a sensory neuropathy that depends on the alteration of the dorsal cutaneous sensory nerves of the upper back. This nerve alteration may be secondary to localized entrapment (possibly by the adjacent muscles) or central damage (related to the pathologies of the spine) or both [6].

In this study, we clinically and radiologically evaluated 45 patients diagnosed with NP. Although the occurrence of NP is not rare, most cases are undiagnosed. Therefore, clinicians should be careful not to overlook this condition. The most common clinical findings of our patients were itching (100%), burning sensation (49%), and hypoesthesia (40%). In addition, a total of 46 spinal pathologies were revealed radiologically in 39 patients with NP (87%). When compared to the control group, there were a significantly higher number of cervical degenerative changes and HNP of the C6–7 segment in the NP group. We intradermally administered lidocaine diluted with saline over a total of three sessions at two-week intervals for the treatment of NP. We achieved successful results related to pain and pruritus.

Pruritus occurs secondary to neuropathic pain, paresthesia, and hyperesthesia, and negatively affects quality of life in patients with NP [3]. Another important clinical finding in this patient group is a well-defined hyperpigmented macule which is not a primary lesion but develops secondary to chronic rubbing and scratching. Chronic itching causes neurogenic release of substance P into the skin, proliferation of epidermal cells, and hyperkeratosis of the skin. A skin biopsy shows non-specific post-inflammatory hyperpigmentation, mild hyperkeratosis, and infiltration of amyloidosis in the papillary dermis [13, 14]. In the current study, persistent itching was present in all of the patients, but hyperpigmented macules were not seen in eight (18%) patients. The typical hyperpigmented patch was not observed in up to two-thirds of the published cases in the literature [15, 16]. There are reported NP cases in which the appearance of the skin is completely normal [17, 18].

Degenerative changes in the spine, osteophytes, trauma, accidents, spinal stenosis, disc herniation, paraspinal muscle spasm, and fibrous bands are the main factors that cause spinal nerve entrapment [13, 19]. Patients with NP frequently have several structural spinal vertebral diseases on the cervical and/or thoracic spine. Degenerative changes, disc herniation, kyphosis, osteoarthritis, scoliosis, spinal stenosis, vertebral arthrosis, and hyperostosis are among the changes in the spine related to NP. Savk et al. evaluated the entire vertebral column radiography and MRI findings of 43 patients with NP. They observed degenerative changes or HNP in 34 patients (79%). A total of 22 HNP were observed in 17 patients. HNP were on the cervical segments in 18 cases and thoracic segments in four. In that study, in 61% of the patients, the localization of symptoms was consistent with the radiological findings, such as degenerative changes. The authors concluded that spinal changes could be considered as a contributing factor to NP [20]. In the current study, we radiologically evaluated 45 patients with NP and detected 46 spinal pathologies in 39 of these patients (87%). Similarly, we found that the HNP of the cervical segments were at a higher rate than those of the thoracic segments in the NP group. In contrast to Savk et al., we also had a control group in our study. We found significantly more cervical degenerative changes and HNP of the C6–7 segment in the NP group than in the control group. Our study involved the evaluation of the radiological findings of the largest number of patients with NP in the literature.

Alai et al. reported a single case with cervical spinal stenosis correlated with the clinical symptoms of NP. They considered that NP might be a cutaneous sign of an underlying degenerative cervical spine disease [21]. We also observed degenerative changes and spinal stenosis mostly on the cervical spine (22%) in the NP group. The HNP of the C6–7 segment were most commonly revealed disc lesions. These findings support the idea that cervical spinal pathologies may be a contributing factor to the pathogenesis of NP.

Eisenberg et al. reported the case of a 76-year-old male with NP. His MRI revealed C4 nerve root impingement, which was associated with clinical symptoms. For the treatment of this case, the cervical epidural steroid injections applied resulted in a nearly complete recovery of symptoms [22].

Raison-Peyron et al. examined 12 NP patients with dorsal spinal X-rays and detected dorsal arthroses in nine cases. In four of six NP patients, successful results were obtained by spinal physiotherapy [15]. The relief achieved in patients through spinal physiotherapy is evidence that the pathogenesis of NP is related to the compression of the spinal nerves. In our patients, the degenerative changes and HNP of the cervical segments were more common than those of the thoracic segments. Thirty-seven cervical degenerative changes or HNP were detected in 39 patients with NP, while only nine patients had thoracic spinal lesions. According to our results, cervical spinal degenerative changes and HNPs may contribute to the pathogenesis of NP.

Pagliarello et al. clinically evaluated 65 patients (female/male: 1.6) with NP and reported that the female gender had worse disease severity. In addition, they found that a higher BMI was associated with a longer disease duration [16]. In our study, the majority of the patients were female (*n* = 39; 87%). In contrast to Pagliarello et al., we found no statistically significant correlation between gender and VAS-pain scores (*r* = − 0.2, *p* = 0.12). We also did not find any significant correlation between BMI and disease duration (*r* = 0.1, *p* = 0.38).

A treatment modality that completely relieves NP symptoms has not yet been established. Topical, intralesional, systemic, peri-neural, and non-pharmacologic approaches have been tested. Topical anesthetics, topical capsaicin, intralesional corticosteroid, and botulinum toxins are the preferred local treatment applications. Gabapentin, oxcarbazepine, amitriptyline, paravertebral local anesthetic blocks, spinal manipulation, physical therapy (transcutaneous electrical nerve stimulation, stretching and strengthening exercises, and cervical traction) are among the other treatment options for NP [6, 10, 23, 24].

Physiotherapy also aims to correct musculoskeletal dysfunctions and has been reported to have sustained benefits in treating NP [11, 25–27]. Zagarella et al. investigated the effects of a 12-week program involving exercises and stretches (three times daily for five to 10 min) on 12 patients with NP. The authors attempted to relieve the sensory neuropathy caused by paraspinal muscle compression and achieved a satisfactory improvement in 11 of 12 patients without any adverse effects [25].

Sahhar et al. reported 20 patients who underwent a six-week physiotherapy program consisting of targeted pressure and manipulation to release muscular spasm and improve thoracic facet and costovertebral joint mobility. Clinical recovery was mentioned in 13/20 patients, with four patients having complete relief without any adverse effects [26]. Due to their easy accessibility, efficacy, and minimal adverse effects, physical therapy modalities and exercises are commonly considered or combined with other treatments in NP.

Surgical decompression can also be considered as an alternative treatment strategy for peripheral nerve entrapment in NP. Williams et al. reported a 33-year-old female diagnosed with the compression of the dorsal branches of the T4 and T5 spinal nerves. The authors confirmed the NP diagnosis by Marcaine blockage to the right of the spinous process at the T4 and T5 levels. The local anesthetic blockage relieved her pain for four to 5 h. Surgical decompression of the dorsal branches of these nerves was performed, and at 4 months after neurolysis, she was symptom-free [28].

Chtompel et al. presented the case of a 50-year-old woman with spinal cord injury caused by an epidural abscess. This patient was diagnosed with NP and treated with intravenous lidocaine for the management of neuropathic pruritus. The authors applied a series of three infusions of lidocaine at two-week intervals at a dose of 1 mg/kg bolus, followed by 4 mg/kg infusion over 1 h. Following the first two infusions, there was no change in the level of neuropathic pain. However, significant relief of itching was noted. They concluded that intravenous lidocaine was effective in reducing neuropathic itching [2].

In the current study, 20 patients in the NP group suffering from pruritus and neuropathic pain were locally administered therapeutic lidocaine, and improvement was observed to sustain for up to 3 months. We obtained successful results in relieving of pain and pruritus of the patients with NP by simple intradermal lidocaine injections. Therefore, we did not consider the need to block the dorsal spinal nerves with a deeper injection method.

Therapeutic local anesthesia (neural therapy) is a modality using injections with local anesthetics for diagnosis and therapy (indications include functional disorders, inflammatory diseases, and acute and chronic pain). The real purpose, though, is not to apply local anesthetics. The generation of targeted stimuli (through the needle) and the selective extinction of other stimuli (through the local anesthetic) affect both the organization of the nervous system and tissue perfusion, thereby disrupting positive feedback actions (vicious circle) in the pain cycle. This treatment modality stimulates the regulatory mechanisms and plastic properties of the vegetative nervous system, primarily in two stages: first, via segmental reflectory processes, and second via the so-called interference field (irritation zone), which may initiate and/or sustain pain and inflammation regardless of the affected segment [29–33].

This study has certain limitations. Firstly, the radiological findings of the patients were evaluated by X-ray and MRI, but their consistency with the clinical findings was not evaluated. Secondly, the patients were not followed up after the third months of lidocaine application. It remains unclear whether the efficacy of lidocaine treatment is durable or continuous injections are required for greater benefit in patients with NP. Thus, further controlled studies with a long-term follow-up are recommended. We also did not have a control group with NP who did not undergo lidocaine treatment. Thirdly, in the current cross-sectional study, it was difficult to draw definitive conclusions concerning the treatment applied. Further prospective, randomized, controlled studies with larger patient groups are needed to elucidate the clinical properties, underlying spinal pathologies, and etiopathogenesis of NP.

## Conclusion

We conclude that cervical degenerative pathologies, especially HNP of the C6–7 segment are possible contributing factors to NP. The relationship between cervical spinal lesions and NP must be further investigated in future randomized controlled trials. Local lidocaine therapy appears to be a safe treatment option for patients with NP, especially in terms of its antipruritic and pain relief effect that lasts for up to 3 months.

## Data Availability

The datasets generated and/or analysed during the current study are not publicly available due to the patients’ privacy but are available from the corresponding author on reasonable request.

## Abbreviations

NP: Notalgia paresthetica
HNP: Herniated nucleus pulposus
BMI: Body mass index
VAS: Visual analog scale
